# Bacterial Community Dynamics in Full-Scale Activated Sludge Bioreactors: Operational and Ecological Factors Driving Community Assembly and Performance

**DOI:** 10.1371/journal.pone.0042524

**Published:** 2012-08-03

**Authors:** Alexis Valentín-Vargas, Gladys Toro-Labrador, Arturo A. Massol-Deyá

**Affiliations:** Department of Biology, University of Puerto Rico at Mayagüez, Mayagüez, Puerto Rico, United States of America; Institute for Genome Sciences, University of Maryland School of Medicine, United States of America

## Abstract

The assembling of bacterial communities in conventional activated sludge (CAS) bioreactors was thought, until recently, to be chaotic and mostly unpredictable. Studies done over the last decade have shown that specific, and often, predictable random and non-random factors could be responsible for that process. These studies have also motivated a “structure–function” paradigm that is yet to be resolved. Thus, elucidating the factors that affect community assembly in the bioreactors is necessary for predicting fluctuations in community structure and function. For this study activated sludge samples were collected during a one-year period from two geographically distant CAS bioreactors of different size. Combining community fingerprinting analysis and operational parameters data with a robust statistical analysis, we aimed to identify relevant links between system performance and bacterial community diversity and dynamics. In addition to revealing a significant β-diversity between the bioreactors’ communities, results showed that the largest bioreactor had a less dynamic but more efficient and diverse bacterial community throughout the study. The statistical analysis also suggests that deterministic factors, as opposed to stochastic factors, may have a bigger impact on the community structure in the largest bioreactor. Furthermore, the community seems to rely mainly on mechanisms of resistance and functional redundancy to maintain functional stability. We suggest that the ecological theories behind the Island Biogeography model and the species-area relationship were appropriate to predict the assembly of bacterial communities in these CAS bioreactors. These results are of great importance for engineers and ecologists as they reveal critical aspects of CAS systems that could be applied towards improving bioreactor design and operation.

## Introduction

Nowadays, biological wastewater treatment plants (WWTPs) are the most common biotechnological application in the world [1]. More than 15,000 WWTPs operate in the United States alone, 75% of which include a secondary biological treatment, processing billions of liters of sewage per day [2]. From the various alternatives of biological treatment systems that exist, conventional activated sludge (CAS) bioreactors are by far the most commonly used secondary treatment technology [3]. Despite of periodic improvements to the technology since its invention almost a century ago [3] and its ubiquitous global application, little is known about the underlying factors controlling the complex dynamics of the microbial populations interacting in the bioreactors and how those dynamic interactions affect the system’s functional stability [4]. Until recently, a major obstacle was that the science behind most of those technology improvements was almost entirely empirical rather than theoretical [3], [5]. Major changes to the design of CAS systems were done predominantly from an engineering perspective, greatly underestimating the importance of microbial communities as an integral component of these biological treatment systems [3], [5]. Thus, many essential aspects regarding the ecology and dynamics of microbial communities within these systems, necessary for a rational improvement of their design and operation, remain unresolved [6].

Recent efforts have focused on improving the treatment process from a bio-ecological perspective, but so far few studies have been able to establish a clear link between the structure and function of microbial communities and the design and operation of the bioreactors [7]. Most of these efforts have failed due to limiting methodology issues. One of these issues is the modeling of full-scale WWTP bioreactors based on studies of lab-scale and pilot-scale bioreactors [8], [9]. These studies have often been misleading and far from mimicking the real conditions observed in full-scale bioreactors, creating a big gap between their theoretical and their practical contributions [10], [11]. Another issue is that many studies had focused on analyzing single bioreactors [12], [13], neglecting from their analysis the effect that niche-specific factors may play in the structure and function of microbial communities [5], [14], . The most notorious, and therefore highly scrutinized, of these issues is culture- and traditional-microscopy-based studies. These studies, aimed to elucidate the diversity of microbes in WWTPs [16]–[19], proved to be unreliable, irreproducible and created erroneous perceptions of the dominant populations in the bioreactors [20]–[23]. They also failed to consider operational and geographical factors on the composition of the communities [24]–[27].

With the development and application of modern culture-independent molecular techniques in ecological studies of wastewater treatment systems [21], [28]–[30], the capacity of researchers to understand the true dynamics of microbial communities in these ecosystems has greatly been improved [11]. However, de los Reyes [31] explains that advanced molecular studies of microbial communities in WWTPs have led to the emergence of a microbial community “structure-function” paradigm that has not yet been fully clarified. Linking changes in system design and operation with the ecological factors controlling community assembly in the bioreactors will be critical in fully clarifying this “structure-function” paradigm and resolving important operational issues, such as: sludge bulking (*e.g.* [32]), poor biochemical removal (*e.g.* [33]), and system instability (*e.g.* [34], [35]); ultimately resulting in more stable and predictable systems [5]. Moreover, the study of CAS systems could provide, in turn, a research platform for developing and validating ecological principles that could be used to predict the behavior of microbial communities in other engineered and natural ecosystems [36]–[38].

Since bacteria are the dominant microbial group in full-scale WWTPs [7], in the current study we aimed to (*i*) assess and compare the dynamics of bacterial communities in two geographically distant full-scale CAS bioreactors treating domestic sewage in a tropical environment and to (*ii*) establish links between the systems’ operational parameters and the behavior of the communities. We also evaluated the outcome of the study by applying well-established ecological theories that could allow us to better predict how microbial communities assemble in these ecosystems. We hypothesized that (*i*) the structure of the bacterial communities in both bioreactors will significantly differ temporally and spatially from each other (high β-diversity) and in response to operational parameters, and that (*ii*) the functional stability of the system can be linked to the dynamics and diversity of the bacterial communities in the bioreactors. We combined culture-independent molecular techniques and a robust multivariate statistical analysis to test these hypotheses and contribute to fulfilling the urgent need for rationally improved biological wastewater treatment technologies.

## Methods

### Description of WWTPs, Sample Collection and Physicochemical Analysis

Two full-scale domestic WWTPs were studied on the island of Puerto Rico. The first plant is the Puerto Rico Aqueduct and Sewers Authority (PRASA) Regional Wastewater Treatment Plant of Mayagüez, located in the coastal town of Mayagüez (18°14′53″N, 67°09′21″W). This plant has four CAS bioreactors with the capacity of processing 106 mega liters of wastewater per day (MLD). Because the plant’s average daily flow (ADF) (∼41 MLD) is below its maximum capacity, it generally has only 2 bioreactors operating simultaneously, alternating them in two-year periods. Since bioreactor #2 was scheduled to start its two-year operating period days prior to the first scheduled sampling, we selected it for our study in order to maintain consistency throughout the sampling period. The second plant is the PRASA Municipal Wastewater Treatment Plant of Adjuntas, located in the central town of Adjuntas (18°09’59”N, 66°43′40″W). This plant has only one CAS bioreactor with the capacity of processing 2.3 MLD, which routinely operates close to capacity. Since the former plant has a higher capacity we refer to it as the HC plant and the latter as the LC (low capacity) plant. Monthly grab samples of activated sludge were collected from the aeration tanks over a 12-month period (February 2007–January 2008). Samples were collected in sterile 50 mL tubes and transported to the lab on ice for immediate processing. Additional samples were collected concurrently from the WWTPs for physiochemical analysis (Table 1). The different parameters were estimated following standard methods for wastewater analysis [39]. All the necessary permits were obtained for the described field study. All necessary permits to enter the wastewater treatment plants (WWTPs) and for the collection of water and activated sludge samples from the systems were granted by Puerto Rico Aqueduct and Sewers Authority (PRASA, the government agency that supervise and operates the studied treatment plants).

**Table 1:** Average values and standard deviations of the measured operational parameter for each WWTP.^a^

Operational Parameters^b^	HC	LC
BOD- Influent (mg L^−1^)	297.50±186.98	173.33±29.90
BOD- Effluent (mg L^−1^)	4.13±1.60	5.18±3.27
BOD Removal (%)	98.33±0.89	96.75±2.53
TSS- Influent (mg L^−1^)	54.33±12.91	162.83±49.54
TSS- Effluent (mg L^−1^)	1.78±1.89	10.81±11.81
TSS Removal (%)	96.75±3.74	92.67±8.28
ADF (MLD)	41.20±4.27	1.88±0.33
HRT (Hours)	7.21±0.65	8.73±1.78
SRT (Days)	10.59±3.69	3.32±1.63
Food:Microbes ratio (F/M)	0.58±0.45	0.09±0.03
pH	6.63±0.37	6.64±0.34
PO_4_^3−^ (mg L^−1^)	103.92±38.60	61.29±37.87
NO_3_^–^N (mg L^−1^)	4.90±2.09	5.54±2.02
Temperature (°C)	29.92±1.00	26.42±0.67

a WWTP: Wastewater Treatment Plant.

b BOD: Biochemical Oxygen Demand; TSS: Total Suspended Solids; ADF: Average Daily Flow; MLD: Mega Liters per Day; HRT: Hydraulic Retention Time; SRT: Solids Retention Time.

### Terminal Restriction Fragment Length Polymorphism (T-RFLP)

Sub-samples of 3 mL from each 50 mL tube of pre-homogenized activated sludge were serially centrifuged at 10,000 g for 5 min in 1.5 mL tubes. The sludge pellets were washed twice in sterile deionized water to reduce the concentration of PCR inhibitors. Total DNA extractions from the pellets were performed using the FastDNA® SPIN Kit for Soil (MP Biomedical, Ohio, USA) following the manufacturer instructions with the following modification: acid-washed crystals of Polyvinylpyrrolidone were added to the Lysis Matrix E tubes at a concentration of 0.1% before vortexing them for 15 min at maximum speed. Extracted DNA purification and quantification was done as described elsewhere [40]. The 16 S rRNA gene of bacteria was PCR amplified in triplicate, using 100 ng of template DNA and the primer set 27F-1392R [41]. The 5′ terminal of the 27F primer was labeled with the infrared dye IRDye® 700 (LI-COR Biosciences, Nebraska, USA). PCR reactions of 50 µL each were prepared as previously described [40], with the addition of 3% DMSO. The PCR cycling parameters were as follows: initial denaturing at 95°C for 5 min followed by 35 cycles of denaturing at 94°C for 1 min, annealing at 53°C for 1 min, extension at 72°C for 2 min, and final extension at 72°C for 8 min. Triplicate PCR products were then pooled and mixed. Three aliquots from each amplicon pool were individually digested with one of the following restriction endonucleases: *Hae*III, *Rsa*I or *Msp*I. Digestion reactions consisted of 150 ng of PCR product, 1.5 µL of 10X buffer, 0.5 µL of the endonuclease (10 U µL^−1^), and water to a final reaction volume of 15 µL. They were incubated for 4 hours at 37°C, followed by an enzyme inactivation step at 65°C for 10 min. A 2 µL aliquot of each digestion was mixed 1∶1 with a 10% dionized formamide IR2 stop solution (LI-COR Biosciences, Nebraska, USA), and then denatured at 94°C for 5 min. Samples were visualized in 6.5% polyacrylamide gels, processed using the DNA Analyzer LI-COR Biosciences 4300 (LI-COR Biosciences, Nebraska, USA) following the manufacturer instructions. The external and the central wells of each gel were loaded with the molecular marker 50–700 bp IRDye® 700.

### Analysis of T-RFLP Profiles

The terminal restriction fragments (TRFs) profiles were analyzed with Gel-Pro Analyzer V4.5 (Media Cybernetics, Maryland, USA). Each individual TRF, considered an independent operational taxonomic unit (OTU), was assigned a hypothetical molecular weight based on the known sizes of the molecular marker. Thus, only TRFs within the range of the molecular marker (50–700 bp) were considered for the analysis. The relative abundance of the OTUs was based on the bands’ signal intensity, observed on the electropherograms as peak height. Each fluorescence signal was standardized by dividing the height of each peak by the sum of all the peak heights in a single sample profile. To accurately compare multiple T-RFLP profiles, the raw matrices of relative abundance were analyzed using two scripts written on *CLISP* (www.clisp.org). The first script, called *PEAKS*, performed a recursive iteration to detect the minimal values that can separate fragment’s peaks from noise baseline based on a modification of the statistical criteria proposed by Abdo *et al.* [42]. The selection of real peaks was based on exclusion of values equal or larger than the median plus three standard deviations (µ+3σ), the calculation and elimination of higher than expected values was done recursively until no larger values could be removed from the data set and the script saved the last calculated value as the threshold to consider real peaks. A new matrix of relative abundance values was constructed by eliminating all values below the threshold determined for each sample. This matrix was subjected to the second script, called *BINNING*, used to group, in a single OTU, TRFs from different samples with similar molecular weights. Even though the electrophoretic displacement of the TRFs was compared with the displacement of the standard of known molecular weight, subtle differences on the bands migration pattern between runs could create small discrepancies in molecular weight between TRFs of a single OTU. Thus, to compare T-RFLP profiles from different gels they first need to be aligned through a process done by grouping TRFs of similar molecular weights from different profiles [43]. The *BINNING* script explored all possible groups of TRFs (or “bins”) and organized individual sets of data in a unified matrix using three criteria: (*i*) fragment size, (*ii*) congruence and (*iii*) number of peaks within a possible OTU. The fragment size criterion established the maximum binning size of an OTU depending on fragment size as suggested for Automatic rRNA Intergenic Spacer Analysis by Brown *et al*. [44]. The congruence criterion discarded binned groups that cluster two or more peaks of the same profile into the same OTU, subdividing the grouping into two or more OTUs. Finally the script counted the number of peaks that belong to each possible OTU and favored larger bins. The *BINNING* script was repeated twice, once creating bins in ascending order of molecular weight and *vice versa*. The bins selected were used to create the final unified matrices (one per enzyme). The size assigned to each TRF in a bin was an average of the molecular weights of the TRFs within that bin. For further details on the CLISP scripts refer to Caro-Quintero [45].

### Statistical Analysis

#### Community structure analysis

The binned matrices were square-root transformed to minimize the impact of highly dominant OTUs and then subject to several statistical analyses to compare the structure of the bacterial communities within and between bioreactors. The dynamics of bacterial communities in both WWTPs were primarily analyzed by non-metric multidimensional scaling (NMDS). Since the distribution of the scatter points in the NMDS ordination diagram may converge on different arrangements depending upon the random initial conditions of the analysis, 110 iterations of the analysis were run. A stress value was calculated to measure the difference between the ranks on the ordination configuration and the ranks in the original similarity matrix for each repetition [46]. An acceptable stress value should be below 0.1. The ordination with the lowest stress was plotted. Analysis of similarity (ANOSIM) and non-parametric multivariate analysis of variance (NPMANOVA) were conducted to test the differences in overall bacterial community structure between the WWTPs and to further confirm the results observed in the NMDS plot. All three analyses were based on similarity matrices calculated with the Bray-Curtis similarity index selected based on its capacity to support abundance data and because it only accounts for TRFs that are present in two profiles (and not those that are absent) as a similarity between them [43]. Given that Bray-Curtis distance measure doesn’t incorporate any form of scaling for abundance data that could dampen the effect of outliers (*e.i.* dominant and rare species contribute equally to the distance matrix) the square-root transformation of the data sets is therefore justified [47].

#### Analysis of diversity

Rényi diversity profiles, used to rank the bacterial communities according to their relative diversity [48], were calculated from the T-RFLP abundance matrices. These profiles are based on a single continuous parameter, known as Rényi’s alpha (α) parameter, which values at the scale of 1, 2 and infinity are proportional to Shannon’s diversity index, Simpson’s diversity index and Berger–Parker diversity index, respectively [49]. The shape of the profiles is an indication of the evenness within the community; the more horizontal a profile is the more evenly distributed the populations are within that community [48]. Following a recommendation made by Kindt and Coe [48], for this analysis we employed the raw data set instead of the square root transformed data set given that the overall interpretation of the profiles should be invariable to such transformation. We also compared the average richness of OTUs per restriction enzyme and conducted a paired *t*-test against the null hypothesis that there was no significant difference between the richness of OTUs detected in each bioreactor throughout the study.

A power law, known as the species-area relationship (*S = cA^z^*), will be applied to define the relationship between the richness of bacterial populations detected in the systems and the size of the bioreactors [26].

#### Relationship between community dynamics and operational parameters

To directly assess the relationship between the structure of the bacterial communities and the operational parameters of the WWTPs, a canonical correspondence analysis (CCA) was carried out. The CCA scaling focused on inter-sample distances to optimize the position of the samples in the ordination diagram, and was performed using square-root transformed abundance data against the operational parameters as explanatory variables. A CCA ordination biplot of the bacterial communities per treatment plant and the environmental variables arranged along the first two ordination axes was generated by constraining the axes to be linear combinations of environmental variable scores [50]. CCA eigenvalues were generated for each canonical axis to estimate how much of the total variability observed between the microbial communities could be explained by their response to the environmental variables. The CCA ordination diagram was interpreted as described by ter Braak and Smilauer [51]. The statistical significance of the CCA analysis was tested by a Monte Carlo permutation test (1000 unrestricted random permutations; *P*<0.05) of residuals from a reduced model against the null hypothesis that bacterial community composition was unrelated to the measured operational parameters [50].

All statistical analyses were carried out with the software PAleontological STatistics V 1.9 [52], except CCA that was carried out with the software CANOCO V 4.5 [51]. Rényi’s diversity profiles were generated with the R package BiodiversityR [48]. The errors associated to the randomness of the statistical methods and the sampling schemes were ignored given the implementation of several tests of statistical significance (e.g. ANOSIM, *t*-test, permutation test) to validate the observed statistical patterns.

## Results

### WWTPs Operational Parameters

**Figure 1:**
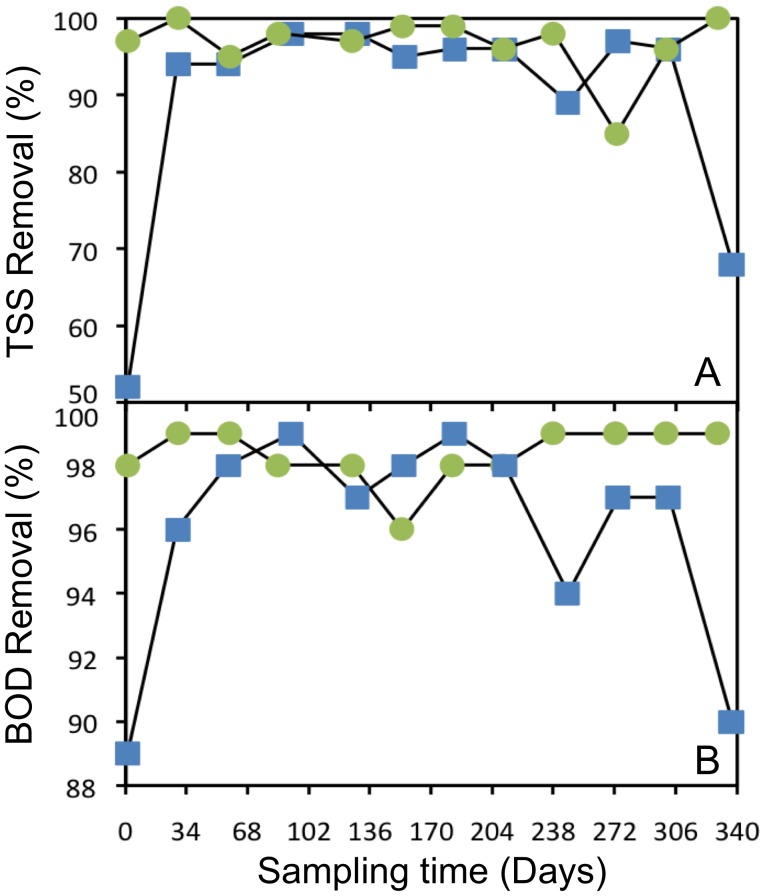
Temporal variations of TSS (A) and BOD (B) removal efficiencies. Percentage values represent the difference between measured influent and effluent concentrations of TSS and BOD for both WWTP: HC (•), LC (▪).

**Figure 2:**
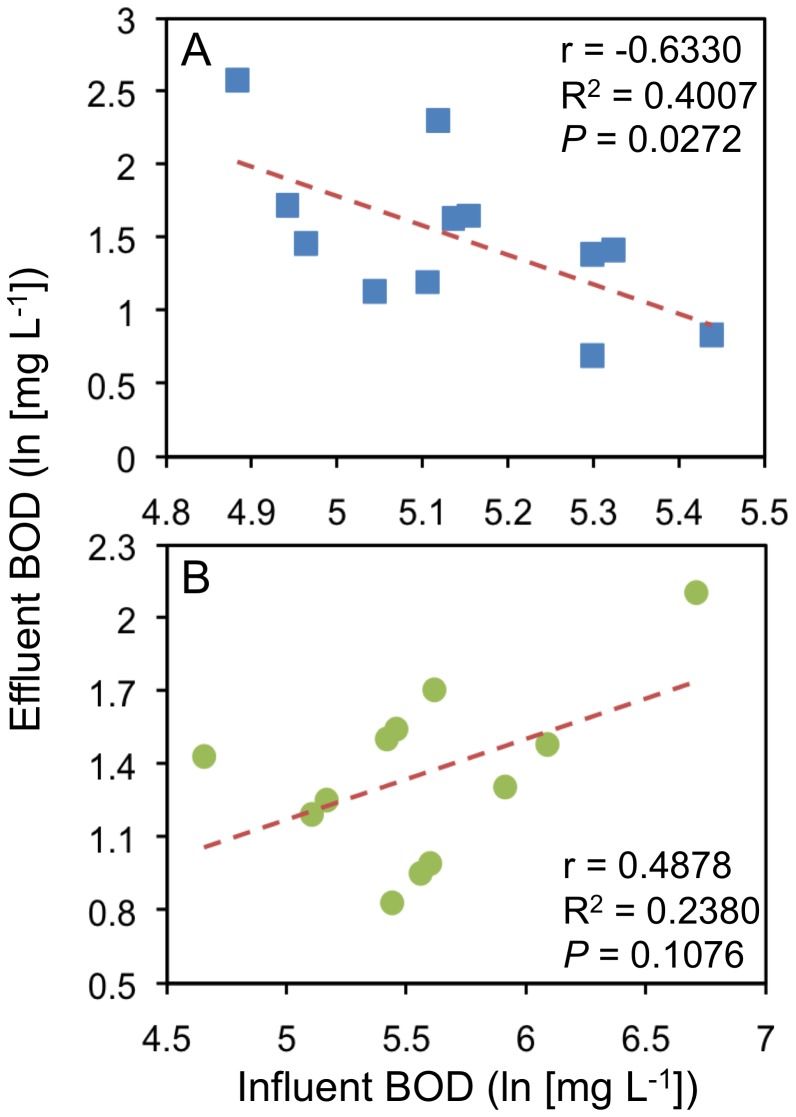
Correlation analyses of the relationship between plants’ influent and effluent BOD concentrations. LC (A) and HC (B). The Pearson’s correlation coefficient (r), the linear regression coefficient of determination (R^2^) and the probability value (*P*) of the analysis are shown.

Details of the operational parameters estimated for both WWTPs are listed in Table 1. Both plants processed domestic sewage year-round under very similar hydraulic retention times (HRT), pH, and fairly constant warm temperatures, typical of the tropical climate in Puerto Rico. The rest of the measured parameters, however, presented important differences between the treatment plants. One of the most obvious differences between them was the average daily flow (ADF), which is directly related to the size of the bioreactors. Throughout the study the ADF in the HC plant was approximately 22 times higher than in the LC plant. On average, the biochemical oxygen demand (BOD) in the influent was much higher in the HC plant (∼72% more) but in spite of these high loads of influent BOD, the HC plant was capable of producing, on average, lower effluent BOD concentration (∼20% less) than the LC plant. Figure 1B shows that the HC plant was more consistent in the removal of BOD from the system, reporting equal or higher BOD removal efficiencies about 75% of the time. One intriguing observation that surfaced during the analysis of the BOD results was that in the LC plant the concentration of the influent BOD seemed to have an adverse effect on the capacity of the plant to remove BOD from the wastewater. Throughout the study, higher levels of influent BOD in the LC plant were consistently associated with lower levels of effluent BOD (higher removal efficiencies), while lower levels of influent BOD were associated with higher levels of effluent BOD (lower removal efficiencies). This was not the case for the HC plant in which lower levels of effluent BOD were, as expected, associated with lower levels of influent BOD. To test this observation we conducted regression and correlation analyses on the relationship between the influent and effluent levels of BOD (Figure 2). The HC plant showed the expected positive relationship between the parameters (r = 0.4878), even though the result from the correlation was not significant (*P* = 0.108). For the LC plant, on the other hand, the analyses showed a strong and significant negative correlation (r = −0.6330, *P* = 0.0272). We ran a Wald test to evaluate the interaction between source bioreactors and influent BOD levels as a predictor variable for the regression model and found that, indeed, the relationship between the parameters significantly differed between plants (*P* = 0.0033).

The influent concentrations of total suspended solids (TSS) throughout the study were, as opposed to those of BOD, higher in the LC plant than in the HC plant. On average, The LC plant received loads of TSS 3 times higher than the HC plant. In terms of the effluent TSS, however, the HC plant was still the leader, producing almost 85% lower effluent concentrations. Although not as evident as for BOD, the HC plant was also more consistent removing TSS (Figure 1A) than the LC plant. With an average SRT of 10.6 days throughout the study, the HC plant showed at least 3 times higher SRT than the LC plant. Likewise, the HC plant showed 6.5 times higher F/M values. In terms of nutrients, both bioreactors showed very similar and rather low concentrations of nitrate-nitrogen. However, the levels of phosphate in both bioreactors were considerably high, with the HC plant showing about 70% higher concentrations.

### Temporal Dynamics of Bacterial Community Structure

**Figure 3:**
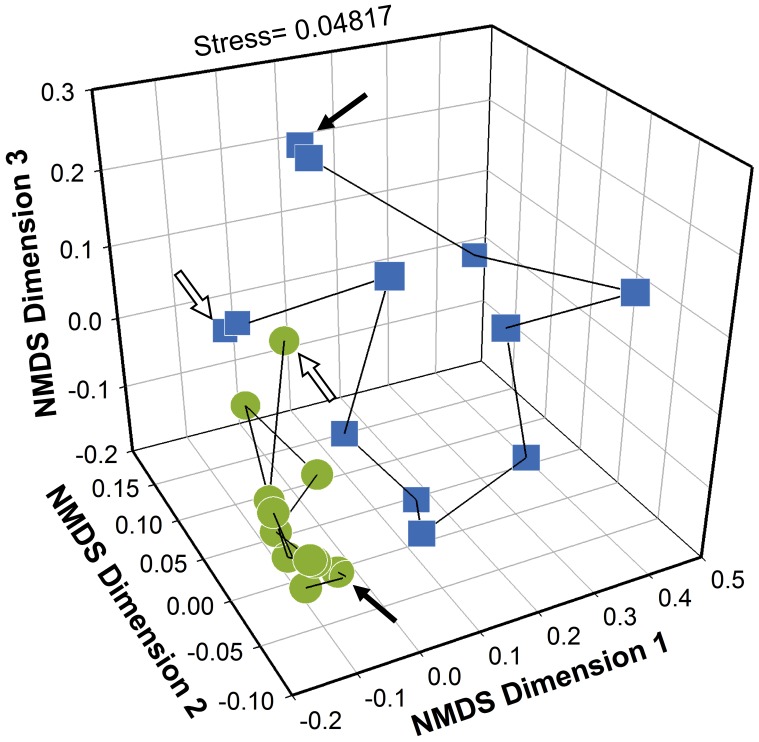
Non-Metric Multi-Dimensional Scaling (NMDS) ordination diagram of temporal variations in bacterial community structure. The ordination is based on a Bray-Curtis similarity matrix of the square-root transformed abundance data obtained from the T-RFLP profiles from both CAS bioreactors: HC (•) and LC (▪). The open arrows point to the first sampling time and the black arrows point to the last sampling time for each bioreactor. Each scatter point in the plot represents the bacterial community in a particular bioreactor at a particular point in time. The separation between the points is relative to their similarity in terms of community composition and they are connected chronologically to show their relative changes throughout the sampling period. The stress value for the tridimensional NMDS ordination is shown. Two non-parametric analyses were calculated to test the significance of the differences observed in the NMDS ordination plot: One-way Analysis of Similarities (ANOSIM): ***R*** **= 0.5451** **(** ***P*** **<0.0001)**; One-way Non-Parametric Multivariate Analysis of Variances (NPMANOVA): ***F*** **= 4.014 (** ***P*** **<0.0001)**.

A NMDS analysis was conducted (Figure 3) to visualize the temporal dynamics of bacterial communities in the CAS bioreactors. Given a stress of 0.04817, the NMDS tridimensional ordination diagram was a reliable representation of the original similarity matrix. In the ordination diagram we can observe that the bacterial community structure in the LC bioreactor was considerably more erratic (*i.e.* dynamic) than the community structure in the HC bioreactor. During the first 4 sampling times of the study the community in the HC bioreactor showed some marked fluctuations, but after the fifth sampling time the community seemed to stabilize and maintain a more consistent yet dynamic structure. It can be also observed from the diagram that the composition of the bacterial communities in both CAS bioreactors substantially diverged from each other. The statistical significance of these differences was confirmed with ANOSIM (*R* = 0.5451, *P*<0.0001) and NPMANOVA (*F* = 4.014, *P*<0.0001).

### Comparison of Bacterial Community Diversity in the CAS Bioreactors

To compare the abundance of populations in the bacterial communities, we analyzed the richness of OTUs as deducted from the T-RFLP profiles (Table 2). On average, 7% more TRFs were detected in the HC community profiles as compared to the LC bioreactor profiles. A paired *t*-test analysis of the bacterial population richness showed that the bacterial community in the HC bioreactor was significantly richer (*P* = 0.0232).

**Table 2:** Comparison of the richness of bacterial populations (OTUs) detected in each CAS bioreactor per restriction enzyme.^a^

WWTP^b^	TRFs Richness^c^				
	*HAE*III (132)	*RSA*I (121)	*MSP*I (130)	Average	
HC	78±8.6	68±11.6	77±12.4	74±10.1	Paired *t*-test
LC	66±10.8	64±15.3	77±8.0	69±12.9	*P* = 0.023

a CAS: Conventional Activated Sludge; OTU: Operational Taxonomic Unit.

b WWTP: Wastewater Treatment Plant.

c TRFs: Terminal Restriction Fragments; Values in parentheses represent the total number of distinctive TRFs detected per restriction enzyme in both bacterial communities combined during the 12 samplings times.

**Figure 4:**
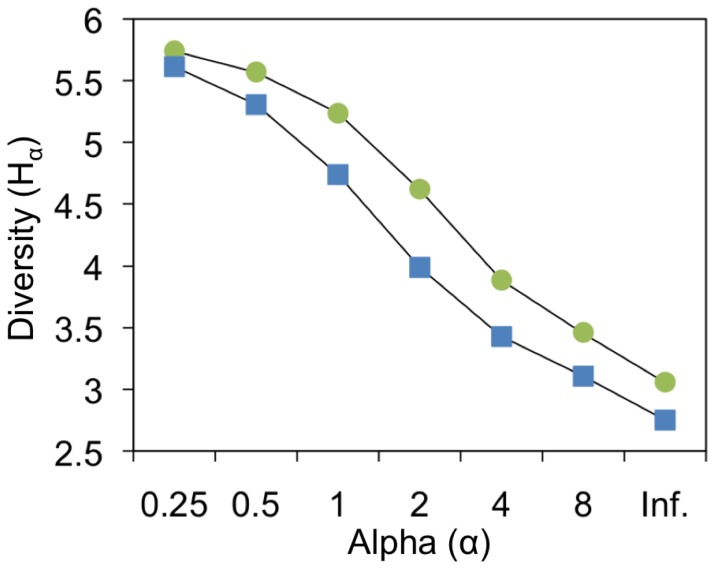
Rényi diversity profiles of the bacterial communities from the CAS bioreactors. The profiles were derived from the T-RFLP raw abundance matrices: HC (•) and LC (▪). The x- and y-axes show the alpha value of the Rényi’s formula and their associated Rényi diversity profile values (H*α*), respectively. Rényi profile values at the scales of 1, 2 and infinite are proportional to Shannon diversity index, Simpson diversity index and Berger–Parker diversity index, respectively (see Kindt and Coe [47] for further information).

In order to rank the bacterial communities according to their diversity, Rényi diversity profiles were generated for each WWTP from the T-RFLP abundance data (Figure 4). Rényi diversity profiling is an ordering technique aimed to easily rank communities of organisms according to their diversity. The selection of indices to compare diversity across ecosystems has often been criticized for being arbitrary and inconsistent since the ranking of communities may change when different indices are used [48]. Defining a family of diversity indices based upon a single continuous parameter is recommended to make the diversity ordering more robust [53]. Thus, ranking based on diversity profiles is ideal because several indices are collectively considered in a single analysis and if the ranking of the communities changes between alpha-values then the profiles are not comparable. Rényi’s diversity ordering method is preferred over other ordering methods [*e.g.* 54,55] because of its superiority on effectively managing a wide range of data sets’ sizes and clearly pointing out the non-comparability between certain profiles [53]. Moreover, as opposed to other methods that are more sensitive to the presence of rare species in the community [*e.g.* 55], Renyi’s method seems to be more sensitive to the presence of the dominant populations and relatively indifferent to the presence of the rare ones [53].

As inferred from the Rényi profiles, the bacterial community from the HC bioreactor was consistently more diverse than the community from the LC bioreactor across all diversity indices represented by the alpha-values. Also, it seems from the shape of the profiles that the populations in the HC bioreactor were more evenly distributed.

### Relationship between Operational Parameters and Bacterial Community Structure

**Figure 5:**
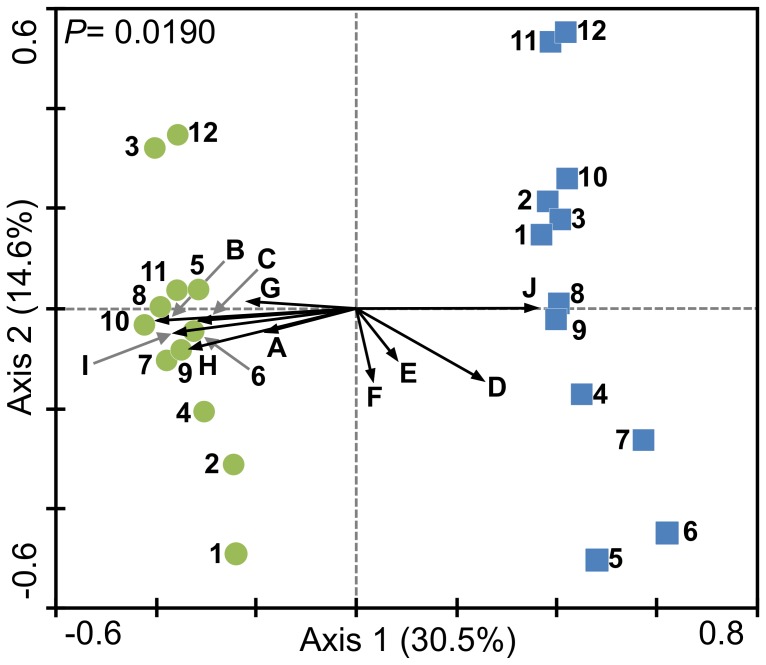
Canonical Correspondence Analysis (CCA) of the relationship between operational parameters and bacterial community structure. The ordination was based on square-root transformed data of the measured operational parameters (arrows): BOD-influent (A), Flow (B), F/M (C), HRT (D), NO_3_^–^N (E), pH (F), PO_4_^3−^ (G), SRT (H), Temperature (I), TSS-influent (J); and the T-RFLP abundance profiles from both CAS bioreactors: HC (•), LC (▪). Numbers next to symbols indicate the relative sampling time. An unrestricted Monte-Carlo permutation test was performed (1000 permutations) to determine the statistical significance of the relationship between the environmental variables and the canonical axes.

A CCA (Figure 5) was conducted to determine which operational parameter had a stronger influence on the assemblage of the bacterial communities in the bioreactors and how the relationship between biotic and abiotic components of the systems translates into the bioreactor’s functional stability. Given a statistically significant CCA ordination (*P* = 0.0190), the null hypothesis of no relationship between bacterial community composition and measured operational parameters was rejected. The CCA eigenvalues for the first two canonical axes showed that the measured operational parameters accounted for approximately 45.1% of the total variability observed in bacterial community structure. From a close examination of the CCA ordination diagram several observations could be made. First, we can appreciate a very prominent separation between samples from different WWTPs into opposite sides of the first canonical axis, meaning that the bacterial communities in each CAS bioreactor responded very differently (entirely opposite most of the time) to the measured operational parameters. We must emphasize that during the CCA analysis no distinction was made between samples from different WWTPs (*i.e.* no nominal variables were used to differentiate samples by their origin). Thus, the separations between the samples from the two plants and the direction of their response to the operational variables should not be an artifact of the analysis but a real ecological trend. Secondly, we can see that most parameters were highly correlated to the first axis, which explains most of the variability in the bacterial communities. From all the parameters, ADF, influent TSS, SRT, F/M, HRT and temperature seem to have the strongest influence on the bacterial communities’ composition in the bioreactors. Although influent BOD and phosphate levels in the bioreactors seemed slightly less influential on the community composition, they were still highly correlated to the first axis. Levels of nitrate-nitrogen and pH in the bioreactors were more related to the differences explained by the second axis and had the weakest influence on the community composition at any given time. Interestingly, very conspicuous parameters (*i.e.* ADF, F/M, SRT) had a strong positive relationship with the bacterial communities from the HC plant and a strong negative relationship with the communities from the LC plant, while other conspicuous parameters (*i.e.* TSS, HRT) behaved the opposite.

## Discussion

In this study, we aimed to assess the dynamics of bacterial communities in two full-scale CAS bioreactors and evaluate the relationship between the WWTP performance and the behavior of the communities, relying primarily on a time series of T-RFLP profiles of community structure. By combining the T-RFLP profiles with a robust statistical analysis we were able to expose differences between the bacterial communities in the bioreactors in terms of composition, diversity and response to operational parameters. We were also able to link the performance of the systems and the mechanisms driving community assembly in the bioreactors. Overall, the two original hypotheses that motivated this study were largely supported by the results.

### Assessment of the Bacterial Alpha- and Beta-diversities in the CAS Bioreactors

The biodiversity of microbial communities in CAS bioreactors is a key factor that needs to be scrutinized as it may correlate with community functional redundancy, and therefore, system functional stability [4], [56], [57]. The two main measurements applicable to spatial-scale biodiversity are: alpha-diversity (α, the diversity of populations within a defined ecosystem) and beta-diversity (β, a comparison of the variations in community composition between two ecosystems). Comparisons of α-diversity are univariate (*e.g.* two samples could have the same species richness but not share any taxa), while β-diversity measures dissimilarity among samples (*i.e*. taxa composition and relative abundance) through multivariate methods [58]. Several reports have shown that standardized T-RFLP profiles processed in parallel can be used to assess both the β-diversity [56], [59] and α-diversity [11], [12], [60] of complex microbial communities.

Our results show that the HC bioreactor had a significantly higher bacterial α-diversity as compared to the LC bioreactor (Table 2, Figure 4), plus, the bacterial communities in both bioreactors had a significantly high β-diversity (Figure 3). Furthermore, the bacterial community in the HC bioreactor was remarkably less dynamic than the bacterial community in the LC bioreactor (Figure 3). These results are truly pertinent if we consider that previous studies suggested that community biodiversity could be positively correlated to system’s function (*i.e.* efficiency and stability) in treatment bioreactors [11], [61] and other engineered ecosystems [34]. Richness analysis of TRFs (Table 2) showed that generally fewer TRFs were detected in the LC system, whereas the variation in the total amount of TRFs detected per sample was greater in this system than in the HC system. This suggests that the community in the LC bioreactor was not only less stable in terms of structure (*i.e.* taxa dominance) but also in terms of taxa richness. Patterns in β-diversity can further be explored by analyzing differences in the abundance of key populations that significantly contribute to the overall dissimilarity between the communities. For instance, a similarity percentage analysis (Table S1) revealed that the *Betaproteobacteria* class was a potentially critical group within the HC bioreactor that significantly contributed to the differences between the communities. This is not surprising, considering that the rather long SRT in the HC plant coincide with the fact that crucial nitrifying *Betaproteobacteria* in WWTPs require extended growing periods (>5 days) in order to build a functionally stable ammonia-oxidizing community [57], [62].

Overall, these results are in line with other reports showing that bacterial diversity is consistently higher in WWTPs with larger capacities [26] and also that geographically distant WWTPs, in spite of their size, usually possess divergent community structures [26], [27], [63]. Van der Gast *et al.* [26] showed that the ecological power law known as the species-area relationship could predict the relationship between the number of bacterial populations in membrane bioreactors and the size of the bioreactors. This relationship is a fundamental ecological concept, first modeled by Arrhenius [64] as a power law: *S = cA^z^*, where *S* is the number of species in a sampled community, *A* is the area (the spatial scale of the observation), *c* is an empirically derived taxon- and niche-specific constant, and *z* is a scaling exponent determined by the slope of the log-log line of the relationship that reflects species turnover. Values of *z* for the distribution of macroorganisms have been shown to differ between continuous (0.12–0.19) and insular habitats (0.2–0.4) [65], [66]. In recent years, this relationship has also been shown to apply to microbial communities in both continuous [67] and insular habitats [38], [68]. Van der Gast *et al.* [26] used a modified version of the species-area power law to use volume instead of area as the spatial scale of the sampled habitat. In our case, we did not know the volume of the bioreactors, thus, instead of volume we used the ADF as an indicator of their size to estimate the species-area relationship for these ecosystems. Surprisingly, the slope of the species-area relationship, or in our case the species-flow relationship, for the CAS bioreactors (*z* = 0.359) landed well within the typical range of *z*-values reported for insular habitats. Moreover, van der Gast *et al.* [26] also observed a positive relationship between the evenness of populations and the size of the bioreactors. Likewise, the evenness of the bacterial populations in the CAS bioreactors, as inferred from the Rényi diversity profiles (Figure 4), was higher in the HC bioreactor. This may imply that larger bioreactors with greater space for bacterial colonization have more niche space available, while smaller bioreactors have fewer vacant niches for colonization, which may potentially allow for a small group of populations to co-dominate in the community [26].

The species-area relationship has been described as an important underlying component of the classic theory of Island Biogeography (IB) [66]. The IB ecological model, conceived by MacArthur and Wilson [66] and further developed by Hubbell [69], is essentially a neutral (*i.e.* all populations are assumed to have equal chances to colonize a niche in the island) dispersal-assembly theory, based on two main assumptions: (*i*) in insular ecosystems the assembly of communities is determined by a dynamic equilibrium between extinction and immigration and (*ii*) the rate of that equilibrium is constrained by the size of the island [70]. The theory also implies that communities inhabiting larger islands will have higher biodiversity and lower rates of immigration and extinction, thus having more stable and efficient community structure [5]. Considering the CAS bioreactors as insular ecosystems, we suggest the dynamic assemblage of their bacterial communities could be predicted by the principles of the IB theory as our results are in agreement with the main features of this equilibrium model: the community in the larger bioreactor is more rich and diverse, and also functionally and structurally more stable. This suggestion is in line with previous reports that have either theoretically [3], [5] or empirically [11], [26], [71], [72] suggested that the complex dynamics of microbial communities in WWTP bioreactors could be explained by the underlying principles of the IB theory. Although the analysis of only two CAS bioreactors may be insufficient to validate the universal applicability of the IB theory to predict microbial dynamics on all CAS systems, it does emphasize the need for more theoretical work aimed to rationally improve their operation and design. The IB theory also implies that islands sharing a common source of new species will have more similar communities than those not sharing a common source. Given that both WWTPs serve different regions of Puerto Rico, the high β-diversity observed between them is not surprising. We believe these differences could also be true for CAS systems geographically distant from the ones we studied. For instance, we generated reference clone libraries using samples from both bioreactors (Figure S1) and they revealed distinctive patterns in the dominant bacterial populations (*e.g.* unusually high detection of non-filamentous *Cyanobacteria*) that do not necessarily correspond with bacterial compositions in WWTPs from other parts of the world [7], [13].

One intriguing observation made from the NMDS diagram (Figure 3) was that the bacterial community structure in the HC bioreactor, despite being steadier than the community structure in the LC bioreactor, showed marked fluctuations during the first 4 samples and then seemed to stabilize through the end of the study. As mentioned above, the bioreactor we chose to monitor at the HC plant started its operational period shortly before the first sampling. This coincidence led us to speculate that the fluctuations in bacterial community structure observed during the first 4 sampling times could be explained by a gradual species succession process through which the community changed towards an “optimal”, more stable structure. Although we did not collect additional samples to corroborate this idea, we believe that the community structure observed during the last 8 samples better represents the dynamics of the bacterial community in the HC plant. Furthermore, we did not find any correlation between these initial community fluctuations and the functional stability of the treatment system nor with the measured operational parameters. These observations are consistent with several reports showing that microbial communities during the start-up period of other types of bioreactors suffered from systematic processes of species succession before reaching relative stability over time [73]–[75], and also that these periods of highly dynamic community structure did not affected the performance of the systems [58], [73].

### Influence of Deterministic, Stochastic and Ecological Factors on Communities’ Dynamics

Two kinds of factors are thought to jointly influence the temporal dynamics of bacterial communities in CAS bioreactors and other engineered ecosystems: deterministic (competition and niche-specific variables) and stochastic (probability of microbial dispersal by random events of colonization/extinction or unpredictable fluctuations in the chemical composition of the influent) [12], [36]. However, there is currently a considerable debate between microbial ecologists on which of the two groups of factors are more important in determining temporal community assembly in engineered ecosystems [72].

As we mentioned above, the analysis of the T-RFLP profiles (Figure 3) showed drastic differences between the communities of both bioreactors in terms of temporal dynamics (*i.e.* changes in community structure). Could these differences be suggesting that the size of the LC bioreactor (as a niche-specific consideration) is exerting over the community a selective pressure strong enough to overshadow the influence of stochastic factors on the temporal structuring of the bacterial assemblages? To address this question, we evaluated the impact of operational factors on the temporal and spatial assembly of bacterial communities in the bioreactors.

BOD is considered the most popular operational parameter for assessing the effectiveness of WWTPs and the impact of their effluent on receiving waters [2]. From the CCA plot (Figure 5) we observed that influent BOD was not as significant as most of the measured parameters on explaining the fluctuations in bacterial community structure in the WWTP. However, we found a significant negative relationship between influent and effluent concentration of BOD in the LC plant, but not in the HC plant (Figure 2). This was despite the fact that, on average, the HC plant received higher and more variable loads of influent BOD (Table 1). Therefore, the results from the regression analysis suggest that the microbial community in the LC bioreactor was significantly and negatively more prone to functional fluctuations by changes in the concentration of influent BOD. This negative relationship can be observed in the relative position of the influent BOD vector and the LC samples in the CCA plot (Figure 5). This may partially explain why the LC plant generally showed lower efficiency removing BOD from the system and higher concentrations of effluent BOD. Nevertheless, we must consider that even though the levels of influent BOD in the LC plant were generally low and steady (Table 1), the BOD removal efficiency of the plant was not as stable. This suggests that the efficiency and structure of the LC community may be more prone to variations from stochastic factors (*e.g.* species immigration, drastic changes in the influent chemical composition) than the HC community. Larger bioreactors with higher biodiversity may provide the advantage of a greater buffering capacity to mitigate the impact of these unpredictable variables. Furthermore, the general response of the LC plant to fluctuations in operational parameters seems to be more unpredictable as the sample points in the CCA plot corresponding to the LC temporal communities are distributed through larger gradients along the first and second axes (Figure 5). Given that CCA asserted that ADF was the main predictor of bacterial community assembly in the CAS bioreactors, we suggest that the magnitude to which deterministic and stochastic factors influence the structure and functional stability of bacterial communities in these bioreactors could be strongly influenced by their capacity. The suggestion that higher population evenness in the HC plant implies greater niche space available for bacterial colonization [26] reinforces the idea that deterministic factors in the HC plant are probably more relevant to the process of bacterial community assembly. The overall observations suggest that the initial bacterial community structure in the HC bioreactor (samples 1–4) may have been heavily influenced by stochastic dynamics while the remaining samples (samples 5–12) suggest a rather stable community structure mostly shaped by deterministic dynamics constrained by the size of the bioreactor. Conversely, throughout the study the bacterial community assembly in the LC plant seems to be consistently dominated by stochastic dynamics.

Several reports have assessed the relative importance of deterministic and stochastic factors as drivers of bacterial community dynamics in treatment bioreactors [12], [26], [71], [72], [76]. Some authors argue that either deterministic or stochastic factors alone are sufficient to explain the dynamics of bacterial communities in WWTPs [36], [77]. Nevertheless, our intuition leads us to believe that both components of the ecosystem should be partially responsible for the temporal and spatial dynamics of these complex communities. The proportion to which both groups of factors influence community assembly in full-scale systems and what components of the ecosystem influence the stability of that proportion, however, remains a matter of debate. Some authors have tested the relative impact of stochastic and deterministic factors in the selection of microbial populations in lab-scale bioreactors, finding that under certain selective pressure deterministic factors drove the assembly of the communities [71], [72]. Conversely, other authors reported that in certain full-scale bioreactors the selective pressure of the systems was not enough to favor a deterministic selection of the community, thus allowing for stochastic dynamics to dominate [12], [26]. In a recent report, Ayarza and Erijman [76] theoretically determined that the relative dominance of stochasticity over deterministic dynamics on microbial community assembly in lab-scale bioreactors was positively correlated to the richness of populations in the system. Our results suggest completely the opposite: a richer community and a larger bioreactor were both positively associated with the dominance of deterministic dynamics on the temporal structuring of the bacterial community and the consequent system stability. These observations may seem to contradict the suggestion made above that the assembly of the communities in the bioreactors could be predicted by the IB equilibrium theory, which is essentially a stochastic model. However, our results do not refute the relevance of stochasticity on community assembly in the bioreactor, in fact, they suggest that a gradual increase in the size of the bioreactors could result on a gradual shift from stochastic dynamics towards a more deterministic selection of the bacterial community structure.

A further analysis of the CCA (Figure 5) revealed other parameters (*i.e.* F/M, HRT, SRT, TSS) that may also significantly contribute to the overall differences between the communities. The F/M ratio for activated sludge systems usually ranges between 0.2–0.6, although for CAS plants between 0.2–0.5 is preferred [2]. F/M values above the preferred range are interpreted as the system has more biodegradable material (*i.e.* BOD) than the microbial communities can optimally handle. Despite the high F/M levels in the HC plant, which can be associated to the contrasting levels of influent BOD and TSS, the system’s SRTs (optimally 5–15 days for CAS systems) provided sufficient time for an efficient and robust community structure to develop. On the other hand, the very low values of F/M in the LC plant suggest that the microbial community in the bioreactor was starving. The low F/M values in the LC plant could be associated to its high levels of influent TSS, justifying its rather short SRTs, and suggesting a high mortality rate among the microbial populations. The latter suggestion could further sustain the idea that the bacterial community in this plant was heavily shaped through stochastic dynamics. Moreover, the short SRTs could explain poor system performance, as it can directly be linked to common operational problems (*e.g.* sludge bulking, poor nutrient removal) [2].

Few reports have looked into the effects of HRT on the assembly of microbial communities in WWTPs [57]. It is evident, however, that fluctuations in HRT (typically 4–8 h for CAS systems) affect substrate gradients in treatment bioreactors; hence, it likely exerts a selective pressure on the microbial communities [57]. Han *et al.* [78] reported that in lab-scale bioreactors shorter HRTs were correlated with less efficient and less diverse microbial communities. This correlation could also be true for the LC plant, which had lower HRT and lower bacterial diversity. Although we do not quite understand the specific effect of HRT on the communities, we believe the current report is the first one to show a strong influence of HRT on bacterial community dynamics in full-scale CAS bioreactors. Further studies will be necessary to clarify the link between the two.

Besides the influence that external factors may have on the dynamic of bacterial communities in the bioreactors, ecological traits intrinsic to the communities themselves could also be crucial to maintain steadier community function and structure over time. The traits of microbial communities that define their capacity to maintain function over time in dynamic ecosystems can be divided in three basic mechanisms: (*i*) resistance of the community to fluctuations in composition, (*ii*) community resiliency after disturbing events, (*iii*) and community functional redundancy [79]. The results from NMDS suggest that the HC community was more resistant despite marked fluctuations in operational parameters, especially during the last 8 months of the study. The slow succession process we saw in the HC plant during the initial months could be attributed to mechanisms of resilience through which the community reached stability after the highly disturbing process it underwent during the bioreactor’s start-up period. Nonetheless, the fact that there seems to be no correlation between the system’s functional stability and the dynamics of the bacterial community during this initial start-up period suggests that functional redundancy rather than resilience was the key ecological trait driving functional stability during that time [80]. According to Briones and Raskin [34], ecosystem stability is not the outcome of population diversity *per se*, but of functional redundancy, ensured by the presence of a reservoir of species able to perform the same ecological function. However, functional redundancy does correlate with community biodiversity [34]. Therefore, by having higher biodiversity, the bacterial community in the HC plant ensured a more stable treatment process even under highly dynamic periods due to its superior functional redundancy. Conversely, results from the LC plant suggest that its community was neither resistant nor resilient. Therefore, the mechanism most likely used by the community to maintain BOD and TSS removal efficiency usually above acceptable levels (*i.e.* >85%) seems to be functional redundancy, although at a level inferior to that observed in the HC plant.

The outcome of the current study can be divided into 4 major findings. (*i*) The bacterial community in the HC bioreactor was more diverse, less dynamic and significantly different from the one in the LC bioreactor. These characteristics were closely linked to the performance of the systems and partially explained by a positive species-flow relationship. (*ii*) Both stochastic and deterministic factors are intricately involved on the assembly of bacterial communities in the system, but the proportion to which either category of factors influence that process seems to be heavily influenced by the biodiversity and capacity of the bioreactor. (*iii*) The mechanisms utilized by the communities to handle the selective pressure of stochastic and deterministic factors and maintain functional stability over time involved a dynamic combination of resistance and functional redundancy. (*iv*) Finally, the principles of the IB ecological model could be appropriate to predict the assemblage and efficiency of the communities. Further work is necessary to see if these patterns hold for full-scale CAS bioreactors with different capacities from other parts of the world and to elucidate the boundaries separating the dominance of either stochastic or deterministic dynamics on community assembly. Furthermore, it will be necessary to translate these observations into process-based mathematical models to be able to better explain and predict the mechanisms driving the dynamics of the bacterial communities in the bioreactors in the light of ecological theory. Given that only 12 time-points may prove to be insufficient to explain the observed patterns by such models, a more exhaustive time-series sampling (*e.g.* weekly samples) is recommended.

This study is of great importance for engineers and microbial ecologists dedicated to optimizing the design of CAS and other treatment systems as it provides evidence linking system performance and operational conditions with the temporal assembly and performance of bacterial communities in the bioreactors. If our findings and suggestions were validated by future studies in other CAS systems of distinctive size and geographical location, they could have deep implications in the way CAS systems are designed and operated. For instance, knowing that larger bioreactors will promote more efficient and stable microbial communities could influence the choice of constructing a single large bioreactor or several small bioreactors to serve a human population [5]. This could be crucial if we consider that the operating cost of WWTPs on a *per capita* basis decreases with increasing reactor size [81]. Our work emphasizes the utility of integrating theoretical ecology in the design and operation of WWTPs as it may ultimately allow for microbial community assembly to become more predictable [3]. This contributes to the increasing pool of evidence suggesting that patterns of microbial assembly and diversity in the environment can be predicted by well-established ecological theories previously thought to apply only to macroorganisms.

## Supporting Information

Figure S1 **Relative abundance of bacterial groups assessed by 16S rRNA environmental clones libraries.** The PCR reactions were carried out using the same primers (without fluorochrome) and protocol applied for the generation of the T-RFLP profiles. PCR products were cloned with the pGEM-T vector system (Promega Corp.) and purified using the Wizard Plus SV DNA Purification System (Promega Corp.). Vector’s inserts were sequenced by the High-Throughput Sequencing Unit (University of Washington, Washington, USA). The phylogenetic affiliation of the sequences was determined using the Sequence Match and Classifier tools available as part of the Ribosomal Data Base project V 10. A total of 192 clones were sequenced, from those, 97 were finally selected for analysis after an extensive process of quality control in which too-short (<400 bp), low-quality and chimerical sequences were eliminated from the data set. The samples used to construct these libraries for both WWTPs plants were collected during the 6th sampling time. Note: HICAP = HC, and LOWCAP = LC.
(PDF)

Table S1 Output of SIMPER analysis showing OTUs responsible for approximately 25% of the overall average dissimilarity (∼54%) observed between the bacterial communities in the CAS bioreactors. Similarity persentage (SIMPER) analysis was conducted to determine which OTUs contributed the most to the average dissimilarity in bacterial community structure observed between the treatment plants. Only those OTUs contributing to the top 25% of the total dissimilarity observed were reported. A putative phylogenetic classification was assigned to each TRF using the Phylogenetic Assignment Tool (PAT+) included in the Microbial Community Analysis III (MiCA 3) web-based software package and compared against “good quality” bacterial sequences (>1200 bp) from the Ribosomal Database Project 10. Out of the 383 distinctive TRFs detected by the three restriction enzymes combined, only 35 (∼9% of the total) were needed to explain 25% of the overall differences (∼54%) observed between the two microbial communities. Also, about 57% of those 35 OTUs were more abundant in the HC bioreactor. From the assignment of putative phylogenies to the TRFs we can deduct that the Phylum that probably contributed the most to the total dissimilarity between the microbial communities was *Firmicutes*. Also, *Betaproteobacteria* and *Deltaproteobacteria* classes were only associated to TRFs from the HC and LC bioreactors, respectively.
(PDF)

## References

[1] Seviour R, Nielsen PH (2010) Microbial ecology of activated sludge. London: IWA Publishing Company. 688 p.

[2] Bitton G (2011) Wastewater microbiology. Hoboken, NJ: John Wiley and Sons. 746 p.

[3] Graham DW, Smith VH (2004) Designed ecosystem services: Application of ecological principles in wastewater treatment engineering. Front Ecol Environ 4: 199–206.

[4] Wang X, Wen X, Yan H, Ding K, Zhao F, et al (2011) Bacterial community dynamics in a functionally stable pilot-scale wastewater treatment plant. Bioresour Technol 102: 2352–2357. 21095118 10.1016/j.biortech.2010.10.095

[5] Curtis TP, Head IM, Graham DW (2003) Theoretical ecology in engineering biology. Environ Sci Technol 37: 64A–70A. 10.1021/es0323493 12630455

[6] Wells GF, Park HD, Eggleston B, Francis CA, Criddle CS (2011) Fine-scale bacterial community dynamics and the taxa–time relationship within a full-scale activated sludge bioreactor. Water Res 45: 5476–5488. 21875739 10.1016/j.watres.2011.08.006

[7] Wagner M, Loy A, Nogueira R, Purkhold U, Lee N, et al (2002) Microbial community composition and function in wastewater treatment plants. Antonie van Leeuwenhoek 81: 665–680. 12448762 10.1023/a:1020586312170

[8] Kaewpipat K, Grady CPL (2002) Microbial population dynamics in laboratory-scale activated sludge reactors. Water Sci Technol 46: 19–27. 12216622

[9] Padayachee P, Ismail A, Bux F (2006) Elucidation of the microbial community structure within a laboratory-scale activated sludge process using molecular techniques. Water SA 32: 679–686.

[10] Jenkins D (2008) From total suspended solids to molecular biology tools- a personal view of biological wastewater treatment process population dynamics. Water Environ Res 80: 677–687. 18751531 10.2175/106143008x276679

[11] Saikaly PE, Stroot PG, Oerther DB (2005) Use of 16S rRNA gene terminal restriction fragment analysis to assess the impact of solids retention time on the bacterial diversity of activated sludge. Appl Environ Microbiol 71: 5814–5822. 16204492 10.1128/AEM.71.10.5814-5822.2005 PMC1265999

[12] Ofiţeru ID, Lunn M, Curtis TP, Wells GF, Criddle CS, et al (2010) Combined niche and neutral effects in a microbial wastewater treatment community. Proc Natl Acad Sci U S A 107: 15345–15350. 20705897 10.1073/pnas.1000604107 PMC2932620

[13] Sanapareddy N, Hamp TJ, Gonzalez LC, Hilger HA, Fodor AA, et al (2009) Molecular diversity of a North Carolina wastewater treatment plant as revealed by pyrosequencing. Appl Environ Microbiol 75: 1688–1696. 19114525 10.1128/AEM.01210-08 PMC2655459

[14] Fierer N, Lennon JT (2011) The generation and maintenance of diversity in microbial communities. Am J Bot 98: 439–448. 21613137 10.3732/ajb.1000498

[15] Nemergut DR, Costello EK, Hamady M, Lozupone C, Jiang L, et al (2011) Global patterns in the biogeography of bacterial taxa. Environ Microbiol 13: 135–144. 21199253 10.1111/j.1462-2920.2010.02315.x PMC5834236

[16] Benedict RG, Carlson DA (1971) Aerobic heterotrophic bacteria in activated sludge. Water Res 5: 1023–1030.

[17] Dias FF, Bhat JV (1964) Microbial ecology of activated sludge. Appl Microbiol 12: 412–417. 14215970 10.1128/am.12.5.412-417.1964 PMC1058146

[18] Lighthart B, Oglesby RT (1969) Bacteriology of an activated sludge wastewater treatment plant: A guide to methodology. J Water Pollut Control Fed 41: R267–R281.

[19] van Veen W (1973) Bacteriology of activated sludge, in particular the filamentous bacteria. Antonie van Leeuwenhoek 39: 189–205. 4578055 10.1007/BF02578852

[20] Eschenhagen M, Schuppler M, Röske I (2003) Molecular characterization of the microbial community structure in two activated sludge systems for the advanced treatment of domestic effluents. Water Res 37: 3224–3232. 14509710 10.1016/S0043-1354(03)00136-2

[21] Snaidr J, Amann R, Huber I, Ludwing W, Schleifer K (1997) Phylogenetic analysis and in-situ identification of bacteria in activated sludge. Appl Environ Microbiol 63: 2884–2896. 9212435 10.1128/aem.63.7.2884-2896.1997 PMC168584

[22] Wagner M, Amann R, Lemmer H, Schleifer K (1993) Probing activated sludge with oligonucleotides specific for proteobacteria: Inadequacy of culture-dependent methods for describing microbial community structure. Appl Environ Microbiol 59: 1520–1525. 8517747 10.1128/aem.59.5.1520-1525.1993 PMC182113

[23] Watanabe K, Yamamoto S, Hino S, Harayama S (1998) Population dynamics of phenol-degrading bacteria in activated sludge determined by gyrB-targeted quantitative PCR. Appl Environ Microbiol 64: 1203–1209. 9546154 10.1128/aem.64.4.1203-1209.1998 PMC106130

[24] Green JL, Bohannan BJM (2006) Spatial scaling of microbial biodiversity. Trends Ecol Evol 21: 501–507. 16815589 10.1016/j.tree.2006.06.012

[25] Martiny JBH, Bohannan BJM, Brown JH, Colwell RK, Fuhrman JA, et al (2006) Microbial biogeography: putting microorganisms on the map. Nat Rev Microbiol 4: 102–112. 16415926 10.1038/nrmicro1341

[26] van der Gast CJ, Jefferson B, Reid E, Robinson T, Bailey MJ, et al (2006) Bacterial diversity is determined by volume in membrane bioreactors. Environ Microbiol 8: 1048–1055. 16689725 10.1111/j.1462-2920.2006.00996.x

[27] Wang X, Wen X, Criddle C, Wells G, Zhang J, et al (2010) Community analysis of ammonia-oxidizing bacteria in activated sludge of eight wastewater treatment systems. J Environ Sci (China) 22: 627–634. 20617742 10.1016/s1001-0742(09)60155-8

[28] Loy A, Daims H, Wagner M (2002) Activated sludge: molecular techniques for determining community composition. In: Bitton G, editor. The Encyclopedia of environmental microbiology. Hoboken, NJ: John Wiley and Sons. 26–43.

[29] Onuki M, Satoh H, Mino T, Matsuo T (2000) Application of molecular methods to microbial community analysis of activated sludge. Water Sci Technol 42: 17–22.

[30] Wilderer PA, Bungartz HJ, Lemmer H, Wagner M, Keller J, et al (2002) Modern scientific methods and their potential in wastewater science and technology. Water Res 36: 370–393. 11827344 10.1016/s0043-1354(01)00220-2

[31] de los Reyes FL III (2010) Challenges in determining causation in structure-function studies using molecular biological techniques. Water Res 44: 4948–4957. 20696455 10.1016/j.watres.2010.07.038

[32] Jones PA, Schuler AJ (2010) Seasonal variability of biomass density and activated sludge settleability in full-scale wastewater treatment systems. Chem Eng J 164: 16–22.

[33] Carvalho G, Lemos PC, Oehmen A, Reis MAM (2007) Denitrifying phosphorus removal: Linking the process performance with the microbial community structure. Water Res 41: 4383–4396. 17669460 10.1016/j.watres.2007.06.065

[34] Briones A, Raskin L (2003) Diversity and dynamics of microbial communities in engineered environments and their implications for process stability. Curr Opin Biotechnol 14: 270–276. 12849779 10.1016/s0958-1669(03)00065-x

[35] Gentile ME, Jessup CM, Nyman JL, Criddle CS (2007) Correlation of functional instability and community dynamics in denitrifying dispersed-growth reactors. Appl Environ Microbiol 73: 680–690. 17142382 10.1128/AEM.01519-06 PMC1800737

[36] Curtis TP, Sloan WT (2006) Towards the design of diversity: stochastic models for community assembly in wastewater treatment plants. Water Sci Technol 54: 227–236. 10.2166/wst.2006.391 16898156

[37] Daims H, Taylor MW, Wagner M (2006) Wastewater treatment: a model system for microbial ecology. Trends Biotechol 24: 483–489. 10.1016/j.tibtech.2006.09.002 16971007

[38] Prosser JI, Bohannan BJ, Curtis TP, Ellis RJ, Firestone MK, et al (2007) The role of ecological theory in microbial ecology. Nat Rev Microbiol 5: 384–392. 17435792 10.1038/nrmicro1643

[39] Eaton AD, Clesceri LS, Rice EW, Greenberg AE (2005) Standard methods for the examination of water and wastewater. Washington, DC: American Public Health Association, American Water Works Association, Water Pollution Control Federation. 1368 p.

[40] Rodríguez-Martínez EM, Pérez EX, Schadt CW, Zhou J, Massol-Deyá AA (2006) Microbial diversity and bioremediation of a hydrocarbon-contaminated aquifer (Vega Baja, Puerto Rico). Int J Environ Res Public Health 3: 292–300. 16968977 10.3390/ijerph2006030036 PMC3807524

[41] Blackwood CB, Marsh T, Kim SH, Paul EA (2003) Terminal restriction fragment length polymorphism data analysis for quantitative comparison of microbial communities. Appl Environ Microbiol 69: 926–932. 12571013 10.1128/AEM.69.2.926-932.2003 PMC143601

[42] Abdo Z, Schüette UME, Bent SJ, Williams CJ, Forney LJ, et al (2006) Statistical methods for characterizing diversity of microbial communities by analysis of terminal restriction fragment length polymorphisms of 16S rRNA genes. Environ Microbiol 8: 929–938. 16623749 10.1111/j.1462-2920.2005.00959.x

[43] Schütte UME, Abdo Z, Bent SJ, Shyu C, Williams CJ, et al (2008) Mini-review: Advances in the use of terminal restriction fragment length polymorphism (T-RFLP) analysis of 16S rRNA genes to characterize microbial communities. Appl Microbiol Biotechnol 80: 365–380. 18648804 10.1007/s00253-008-1565-4

[44] Brown MV, Schwalbach MS, Hewson I, Fuhrman JA (2005) Coupling 16S-ITS rDNA clone libraries and automated ribosomal intergenic spacer analysis to show marine microbial diversity: development and application to a time series. Environ Microbiol 7: 1466–1479. 16104869 10.1111/j.1462-2920.2005.00835.x

[45] Caro-Quintero A (2009) Diversity and microbial community structure at a former military range in Vieques (Puerto Rico). MS thesis: University of Puerto Rico, Mayagüez, Puerto Rico. Available: http://search.proquest.com/docview/305078033. Accessed 2012 Jul 11.

[46] Ramette A (2007) Mini-review: Multivariate analyses in microbial ecology. FEMS Microbiol Ecol 62: 142–160. 17892477 10.1111/j.1574-6941.2007.00375.x PMC2121141

[47] Legendre P, Legendre L (1998) Numerical ecology. Amsterdam: Elsevier. 853 p.

[48] Kindt R, Coe R (2005) Tree diversity analysis: A manual and software for common statistical methods and biodiversity studies. Nairobi: World Agroforestry Centre. 196 p.

[49] Jesus ED, Marsh TL, Tiedje JM, Moreira FMD (2009) Changes in land use alter the structure of bacterial communities in Western Amazon soils. ISME J 3: 1004–1011. 19440233 10.1038/ismej.2009.47

[50] ter Braak CJF, Wiertz J (1994) On the statistical analysis of vegetation change: A wetland affected by water extraction and soil acidification. J Veg Sci 5: 361–372.

[51] ter Braak CJF, Smilauer P (2002) CANOCO reference manual and CanoDraw for Windows user’s guide: Software for canonical community ordination- Version 4.5. New York: Microcomputer Power. 500 p.

[52] Hammer Ø, Harper DAT, Ryan PG (2001) PAST: Paleontological statistics software package for education and data analysis. Palaeontol Electron 4: 1–9.

[53] Tothmeresz B (1995) Comparison of different methods for diversity ordering. J Veg Sci 6: 283–290.

[54] Hill MO (1973) Diversity and evenness: a unifying notation and its consequences. Ecol 54: 427–432.

[55] Patil GP, Taillie C (1982) Diversity as a concept and its measurement. J Am Stat Ass 77: 548–567.

[56] Wang X, Wen X, Criddle C, Yan H, Zhang Y, et al (2010) Bacterial community dynamics in two full-scale wastewater treatment systems with functional stability. J Appl Microbiol 109: 1218–1226. 20477893 10.1111/j.1365-2672.2010.04742.x

[57] Yuan ZG, Oehmen A, Peng YZ, Ma Y, Keller J (2008) Sludge population optimization in biological nutrient removal wastewater treatment systems through on-line process control: A review. Rev Environ Sci Biotechnol 7: 243–254.

[58] Palacios C, Zettler E, Amils R, Amaral-Zettler L (2008) Contrasting microbial community assembly hypotheses: A reconciling tale from the Río Tinto. PLoS ONE 3: e3853. 19052647 10.1371/journal.pone.0003853 PMC2587236

[59] Culman SW, Gauch HG, Blackwood CB, Thies JE (2008) Analysis of TRFLP data using analysis of variance and ordination methods: A comparative study. J Microbiol Methods 75: 55–63. 18584903 10.1016/j.mimet.2008.04.011

[60] Matsuda M, Inoue D, Anami Y, Tsutsui H, Sei K, et al (2010) Comparative analysis of DNA-based microbial community composition and substrate utilization patterns of activated sludge microorganisms from wastewater treatment plants operated under different conditions. Water Sci Technol 61: 2843–2851. 20489257 10.2166/wst.2010.212

[61] Miura Y, Hiraiwa MN, Ito T, Itonaga T, Watanabe Y, et al (2007) Bacterial community structures in MBRs treating municipal wastewater: Relationship between community stability and reactor performance. Water Res 41: 627–637. 17184810 10.1016/j.watres.2006.11.005

[62] Kim YM, Cho HU, Lee DS, Park D, Park JM (2011) Influence of operational parameters on nitrogen removal efficiency and microbial communities in a full-scale activated sludge process. Water Res 45: 5785–5795. 21924454 10.1016/j.watres.2011.08.063

[63] Forney LJ, Liu WT, Guckert JB, Kumagai Y, Namkung E, et al (2001) Structure of microbial communities in activated sludge: potential implications for assessing the biodegradability of chemicals. Ecotoxicol Environ Saf 49: 40–53. 11386714 10.1006/eesa.2001.2034

[64] Arrhenius O (1921) Species and area. J Ecol 9: 95–99.

[65] Connor EF, McCoy ED (1979) The statistics and biology of the species-area relationship. Am Nat 13: 791–833.

[66] MacArthur RH, Wilson EO (1967) The theory of island biogeography. Princeton, NJ: Princeton University Press. 203 p.

[67] Horner-Devine MC, Lage M, Hughes JB, Bohannan BJM (2004) A taxa-area relationship for bacteria. Nat 432: 750–753. 10.1038/nature03073 15592412

[68] Bell T, Ager D, Song JI, Newman JA, Thompson IP, et al (2005) Larger islands house more bacterial taxa. Sci 308: 1884. 10.1126/science.1111318 15976296

[69] Hubbell SP (2001) The unified neutral theory of biodiversity and biogeography. Princeton, NJ: Princeton University Press. 448 p.

[70] van der Gast CJ (2008) Chapter 6: Islands shaping thought in microbial ecology. In: Laskin AI, Sariaslani S, Gadd GM, editors. Advances in applied microbiology, Volume 64. Waltham, MA: Academic Press. 167–182. 10.1016/S0065-2164(08)00406-1 18485285

[71] Manefield M, Whiteley A, Curtis T, Watanabe K (2007) Influence of sustainability and immigration in assembling bacterial populations of known size and function. Microb Ecol 53: 348–354. 17264996 10.1007/s00248-006-9167-0

[72] van der Gast CJ, Ager D, Lilley AK (2008) Temporal scaling of bacterial taxa is influenced by both stochastic and deterministic ecological factors. Environ Microbiol 10: 1411–1418. 18205822 10.1111/j.1462-2920.2007.01550.x

[73] Dollhopf SL, Hashsham SA, Tiedje JM (2001) Interpreting 16S rDNA T-RFLP data: Application of self-organizing maps and principal component analysis to describe community dynamics and convergence. Microb Ecol 42: 495–505. 12024232 10.1007/s00248-001-0027-7

[74] Hiraishi A, Narihiro T, Yamanaka Y (2003) Microbial community dynamics during start-up operation of flowerpot-using fed-batch reactors for composting of household biowaste. Environ Microbiol 5: 765–776. 12919412 10.1046/j.1462-2920.2003.00473.x

[75] Hoshino T, Terahara T, Yamada K, Okuda H, Suzuki I, et al (2006) Long-term monitoring of the succession of a microbial community in activated sludge from a circulation flush toilet as a closed system. FEMS Microbiol Ecol 55: 459–470. 16466385 10.1111/j.1574-6941.2005.00047.x

[76] Ayarza JM, Erijman L (2011) Balance of neutral and deterministic components in the dynamics of activated sludge floc assembly. Microb Ecol 61: 486–495. 20972561 10.1007/s00248-010-9762-y

[77] Saikaly PE, Oerther DB (2004) Bacterial competition in activated sludge: theoretical analysis of varying solids retention times on diversity. Microb Ecol 48: 274–284. 15116279 10.1007/s00248-003-1027-6

[78] Han H, Zhang Y, Cui C, Zheng S (2010) Effect of COD level and HRT on microbial community in a yeast-predominant activated sludge system. Bioresour Technol 101: 3463–3465. 20096563 10.1016/j.biortech.2009.12.121

[79] Allison SD, Martiny JBH (2008) Colloquium paper: Resistance, resilience, and redundancy in microbial communities. Proc Natl Acad Sci U S A 105(Supplemental-1): 11512–11519. 18695234 10.1073/pnas.0801925105 PMC2556421

[80] Mills AL, Herman JS, Hornberger GM, Ford RM (2003) Functional redundancy promotes functional stability in diverse microbial bioreactor communities. SAE International, paper number 2003–01–2509. Available: http://www.evsc.virginia.edu/~alm7d/pubs/82-Mills-ICES-03.pdf. Accessed 2012 Jul 15..

[81] Balmér P, Mattsson B (1994) Wastewater treatment plant operation costs. Wat Sci Technol 30: 7–15.

