# Supplementary figures and images for: Bacterial Community Dynamics in Full-Scale Activated Sludge Bioreactors: Operational and Ecological Factors Driving Community Assembly and Performance
Source: PLoS One. 2012 Aug 3;7(8):e42524. doi: 10.1371/journal.pone.0042524 (PMC3411768; doi:10.1371/journal.pone.0042524)

Figure S1.

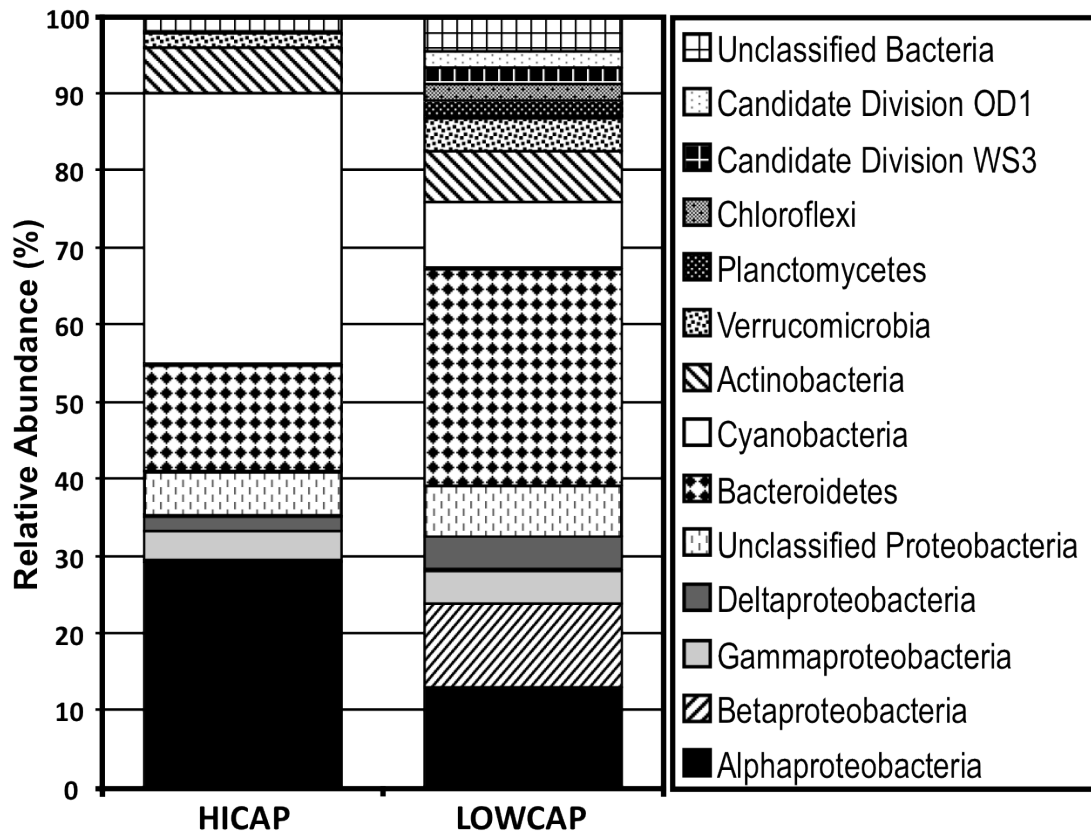

Supplement: Figure S1 — Relative abundance of bacterial groups assessed by 16S rRNA environmental clones libraries. The PCR reactions were carried out using the same primers (without fluorochrome) and protocol applied for the generation of the T-RFLP profiles. PCR products were cloned with the pGEM-T vector system (Promega Corp.) and purified using the Wizard Plus SV DNA Purification System (Promega Corp.). Vector’s inserts were sequenced by the High-Throughput Sequencing Unit (University of Washington, Washington, USA). The phylogenetic affiliation of the sequences was determined using the Sequence Match and Classifier tools available as part of the Ribosomal Data Base project V 10. A total of 192 clones were sequenced, from those, 97 were finally selected for analysis after an extensive process of quality control in which too-short (<400 bp), low-quality and chimerical sequences were eliminated from the data set. The samples used to construct these libraries for both WWTPs plants were collected during the 6th sampling time. Note: HICAP = HC, and LOWCAP = LC. (PDF) [file pone.0042524.s001.pdf]
