# Supplementary material for: Bacterial Community Dynamics in Full-Scale Activated Sludge Bioreactors: Operational and Ecological Factors Driving Community Assembly and Performance
Source: PLoS One. 2012 Aug 3;7(8):e42524. doi: 10.1371/journal.pone.0042524 (PMC3411768; doi:10.1371/journal.pone.0042524)
Supplement: Table S1 — Output of SIMPER analysis showing OTUs responsible for approximately 25% of the overall average dissimilarity (∼54%) observed between the bacterial communities in the CAS bioreactors. Similarity persentage (SIMPER) analysis was conducted to determine which OTUs contributed the most to the average dissimilarity in bacterial community structure observed between the treatment plants. Only those OTUs contributing to the top 25% of the total dissimilarity observed were reported. A putative phylogenetic classification was assigned to each TRF using the Phylogenetic Assignment Tool (PAT+) included in the Microbial Community Analysis III (MiCA 3) web-based software package and compared against “good quality” bacterial sequences (>1200 bp) from the Ribosomal Database Project 10. Out of the 383 distinctive TRFs detected by the three restriction enzymes combined, only 35 (∼9% of the total) were needed to explain 25% of the overall differences (∼54%) observed between the two microbial communities. Also, about 57% of those 35 OTUs were more abundant in the HC bioreactor. From the assignment of putative phylogenies to the TRFs we can deduct that the Phylum that probably contributed the most to the total dissimilarity between the microbial communities was Firmicutes. Also, Betaproteobacteria and Deltaproteobacteria classes were only associated to TRFs from the HC and LC bioreactors, respectively. (PDF) [file pone.0042524.s002.pdf]

Table S1. Output of SIMPER analysis showing OTUs responsible for approximately 25% of the overall average dissimilarity (~54%) observed between the bacterial communities in the CAS bioreactors[a]

TRF (bp)[b]	Cum %[c]	Mean abundance[d]		Restriction enzyme			Putative Phylogeny[e]
		HC	LC	*Hae*III	*Rsa*I	*Msp*I	
242	1.40	0.73	**2.86**	X			*Bacteroidetes, Firmicutes*
245	2.67	1.77	**2.37**		X		*Deltaproteobacteria*
230	3.82	0.00	**2.44**			X	*Firmicutes*
486	4.86	**3.04**	1.14			X	Various groups
239	5.83	0.87	**1.69**		X		*Alphaproteobacteria*
240	6.78	**1.54**	1.36	X			*Firmicutes*
494	7.59	**1.58**	1.33			X	*Cyanobacteria, Gammaproteobacteria*
233	8.36	**1.70**	0.17	X			*Firmicutes*
63	9.10	**1.50**	1.33		X		*Bacteroidetes*
470	9.84	**3.67**	3.08		X		Various groups
477	10.55	**2.27**	1.46		X		Various groups
225	11.26	0.85	**1.36**			X	*Deltaproteobacteria*, *Firmicutes*
251	11.97	0.68	**1.51**		X		*Deltaproteobacteria*
68	12.67	0.84	**1.32**	X			*Actinobacteria*
539	13.37	**1.66**	0.87			X	*Bacteroidetes, Firmicutes*
237	14.05	**1.18**	0.65	X			*Firmicutes*
245	14.73	**0.94**	0.89			X	*Alphaproteobacteria*
491	15.40	**2.09**	1.48			X	*Betaproteobacteria*
545	16.06	2.34	**2.52**			X	*Bacteroidetes, Firmicutes*
68	16.71	0.46	**1.56**		X		*Gammaproteobacteria*
60	17.35	0.75	**1.35**	X			*Gammaproteobacteria*
110	17.97	1.08	**1.50**		X		*Alphaproteobacteria*
227	18.57	**1.74**	1.10	X			*Actinobacteria*
64	19.14	**1.20**	0.72			X	*Firmicutes*
198	19.70	**1.18**	0.96	X			*Betaproteobacteria*
308	20.25	1.01	**1.24**	X			*Firmicutes*
500	20.79	**1.47**	1.46			X	*Alphaproteobacteria*
64	21.33	**1.13**	0.30	X			*Actinobacteria, Alphaproteobacteria*
597	21.87	**1.19**	0.16			X	Various groups
426	22.41	0.56	**1.24**		X		*Cyanobacteria, Deltaproteobacteria*
457	22.94	0.67	**1.02**		X		*Actinobacteria, Firmicutes*
175	23.46	**0.82**	0.42		X		*Bacteroidetes, Firmicutes*
257	23.98	**1.10**	0.70	X			*Gammaproteobacteria, Actinobacteria*
218	24.49	**1.56**	1.45	X			Various groups
71	24.99	0.35	**1.13**			X	Various groups

[a]SIMPER: Similarity Percent; OTU: Operational Taxonomic Unit; CAS: Conventional Activated Sludge
[b]TRF (bp): Terminal Restriction Fragments identified by their size in base pairs
[c]Cum %: Cumulative percent of contribution to the overall dissimilarity between groups
[d]Values in bold show in which CAS bioreactor that taxa (population) was more abundant
[e]Putative phylogeny determined with the Phylogenetic Assigment Tool (PAT+); Various Groups: 3 or more phylogenetic groups (Phyla) were associated to this TRF
